# The MoxR ATPase RavA and Its Cofactor ViaA Interact with the NADH:Ubiquinone Oxidoreductase I in *Escherichia coli*

**DOI:** 10.1371/journal.pone.0085529

**Published:** 2014-01-15

**Authors:** Keith S. Wong, Jamie D. Snider, Chris Graham, Jack F. Greenblatt, Andrew Emili, Mohan Babu, Walid A. Houry

**Affiliations:** 1 Department of Biochemistry, University of Toronto, Toronto, Ontario, Canada; 2 Department of Biochemistry, Research and Innovation Centre, University of Regina, Regina, Saskatchewan, Canada; 3 Banting and Best Department of Medical Research, Terrence Donnelly Centre for Cellular and Biomolecular Research, University of Toronto, Toronto, Ontario, Canada; Universitat Pompeu Fabra, Barcelona Research Park of Biomedicine (PRBB), Spain

## Abstract

MoxR ATPases are widespread throughout bacteria and archaea. The experimental evidence to date suggests that these proteins have chaperone-like roles in facilitating the maturation of dedicated protein complexes that are functionally diverse. In *Escherichia coli*, the MoxR ATPase RavA and its putative cofactor ViaA are found to exist in early stationary-phase cells at 37°C at low levels of about 350 and 90 molecules per cell, respectively. Both proteins are predominantly localized to the cytoplasm, but ViaA was also unexpectedly found to localize to the cell membrane. Whole genome microarrays and synthetic lethality studies both indicated that RavA-ViaA are genetically linked to Fe-S cluster assembly and specific respiratory pathways. Systematic analysis of mutant strains of *ravA* and *viaA* indicated that RavA-ViaA sensitizes cells to sublethal concentrations of aminoglycosides. Furthermore, this effect was dependent on RavA's ATPase activity, and on the presence of specific subunits of NADH:ubiquinone oxidoreductase I (Nuo Complex, or Complex I). Importantly, both RavA and ViaA were found to physically interact with specific Nuo subunits. We propose that RavA-ViaA facilitate the maturation of the Nuo complex.

## Introduction

The MoxR family of AAA+ ATPases is widespread across different bacterial and archaeal species [1], [2]. Based on sequence similarity and local genetic structure, MoxR proteins are subdivided into seven subfamilies: MRP (MoxR Proper), APE0892, RavA, CGN (CbbQ/GvpN/NorQ), APE2220, PA2707, and YehL [1]. The exact roles of MoxR proteins *in vivo* are unclear, although the experimental evidence collected to date suggests that they have chaperone-like functions and are involved in the maturation and activation of specific protein complexes. For example, MoxR of the MRP subfamily in *Paracoccus denitrificans* and *Methylobacterium extorquens* is important for the activation of methanol dehydrogenase (MDH) [3], [4]. NirQ/NorQ, which belong to the CGN subfamily, are necessary for the activity of nitric oxide reductase in *Pseudomonas stutzeri* [5], *Pseudomonas aeruginosa* [6], *Paracoccus denitrificans* [7], and *Rhodobacter sphaeroides 2.4.3* [8]. In the chemolithoautotrophic eubacterium *Oligotropha carboxidovorans* OM5, CoxD, a member of the APE2220 subfamily, is required for the assembly of the [CuSMoO_2_] cluster in the carbon-monoxide (CO) dehydrogenase, which enables the bacteria to utilize CO as a sole carbon source [9].

MoxR proteins also have important roles in other biological processes. For example, in *Rhizobium leguminosarum*, RL3499 of the MRP subfamily is optimally expressed in stationary phase cells and is important for both membrane integrity and cell morphology [10]. In the crenarachaeal *Acidianus* two-tailed virus (ATV), p618 of the RavA subfamily interacts with p892, which forms filamentous structures and is believed to play a role in the extracellular, host-independent formation of viral tails [11]. In *Francisella tularensis*, the MRP protein, FTL_2000, has been implicated in multiple stress tolerance pathways and was shown to be important for infection [12], [13].

Generally, MoxR proteins co-occur with at least one cofactor that carries a von Willebrand factor A (VWA) domain. The genes encoding these proteins are usually in close proximity within the genome [1]. The VWA domain contains a metal-binding motif, known as the MIDAS (metal ion-dependant adhesion site) motif. This motif binds a single divalent metal cation, usually Mg^2+^, and is often involved in mediating protein-protein interactions [14]. While eukaryotic VWA proteins have been characterized extensively, the cellular function of prokaryotic VWA proteins remains poorly understood. Current experimental evidence for these proteins suggests diverse functions, including surface adhesion, fibrinogen binding, metal insertion into protoporphyrin IX, and pathogenesis [15]–[18].

Two MoxR proteins are encoded in the genome of *Escherichia coli* K-12 MG1655: RavA (Regulatory ATPase variant A) of the RavA subfamily, and YehL of the YehL subfamily. We have characterized RavA extensively using various biochemical and biophysical methods. RavA co-occurs with the VWA protein ViaA (VWA interacting with AAA+ ATPase), and the genes encoding these proteins form an operon [19]. Under aerobic conditions, the co-expression of RavA and ViaA is primarily dependent on the stationary phase sigma factor σ^S^ (RpoS) [19]. RavA interacts physically with ViaA, which results in the enhancement of RavA ATPase activity [19]. Typical of AAA+ ATPases, RavA forms a hexamer via its AAA+ module [19], [20] as observed based on the X-ray crystal structure we solved for RavA protomer and the 3D electron microscopy reconstruction of the protein hexamer [20]. We also found that RavA interacts strongly with the inducible lysine decarboxylase LdcI (or CadA), forming a large cage-like complex [19], [20]. LdcI is an important acid stress response protein in *E. coli* [21], [22].

Despite the detailed biochemical and biophysical characterization described above, the cellular function of RavA *in vivo* remains elusive. Association of RavA with LdcI suggests a potential role for the AAA+ ATPase in bacterial acid stress response. Recently, we discovered that LdcI binds the alarmone ppGpp, the primary activator of the stringent response [23], and that the binding inhibits LdcI activity [22]. Furthermore, RavA was found to antagonize the effect of ppGpp inhibition on LdcI [20]. While RavA and, indirectly, ViaA might function to modulate the activity of LdcI, we suspect that the system must have other roles in the cell.

To identify other cellular roles for the RavA-ViaA chaperone-like system, we carried out genome wide genetic interaction and microarray analyses, phenotypic screens, and physical interaction studies. These experiments demonstrated that both RavA and ViaA interact with specific subunits of the highly conserved NADH:ubiquinone oxidoreductase I complex (i.e. Nuo complex, or Complex I), particularly with NuoA and NuoF under aerobic conditions, and with the fused NuoCD under anaerobic conditions. To our knowledge, this is the first report of an interaction between the Nuo complex and a member of the MoxR AAA+ ATPases.

## Materials and Methods

### Bacterial strains and plasmids used

All bacterial strains used are listed in Table 1 with the exception of the 30 BW25113 single-gene knockouts (KO) used in our suppression mutation analysis (see below). Wild type (WT) *E. coli* K-12 MG1655 was obtained from ATCC (catalog number 700926). The corresponding single KO mutants for *ravA* (*ΔravA*::*cat*) and *viaA* (*ΔviaA*::*cat*) were generated by transducing the required chloramphenicol resistance KO cassettes (*cat*) from the original DY330 strains to MG1655 via P1 phage [24] as previously described [19]. A double KO mutant for *ravA* and *viaA* (*ΔravAviaA*::*cat*) was also generated in the same manner. The required *cat* KO cassette was generated by PCR using the primers RKO_forward (5′-agaaacgtctatactcgcaatttacgcagaacttttgacgaaagggtgtaggctggagctgcttc-3′) and VKO_reverse (5′-gcgagagcgtcccttctctgctgtaataatttatcgccgccagcgcatatgaatatcctccttag-3′), and the pKD3 template plasmid as described [19]. The *cat* KO cassettes in *ΔravA*::*cat*, *ΔviaA*::*cat* and *ΔravAviaA*::*cat* was later removed using the pCP20 plasmid that expresses the FLP recombinase [25] to obtain *ΔravA*, *ΔviaA* and *ΔravAviaA*, respectively, with no markers. The generated strains were verified by sequencing. Only KOs without markers (clean KOs) were used in the subsequent experiments with the exception of the microarray experiments.

**Table 1:** List of bacterial strains and plasmids used in this study.

Bacterial Strains	Genotype	Reference
MG1655	F-, rph-1, λ-	[75]
MG1655 *ΔravA*::*cat* *	MG1655, *ΔravA*::*cat*	[19]
MG1655 *ΔviaA*::*cat*	MG1655, *ΔviaA*::*cat*	[19]
MG1655 *ΔravA*	MG1655, *ΔravA*	This paper
MG1655 *ΔviaA*	MG1655, *ΔviaA*	This paper
MG1655 *ΔravAviaA*	MG1655, *ΔravAviaA*	This paper
DY330	W3110, *ΔlacU169*, *gal490*, *λcI857*, *Δ(cro-bioA)*	[76]
DY330 *ΔravA*::*cat* *	DY330, *ΔravA*::*cat*	[19]
DY330 *ΔviaA*::*cat*	DY330, *ΔviaA*::*cat*	[19]
DY330 *ΔravA-viaA*::*cat*	DY330, *ΔravAviaA*::*cat*	This paper
DY330 *nuoA-SPA*::*kan*	DY330, *nuoA-SPA*::*kan*	[77]
DY330 *nuoB-SPA*::*kan*	DY330, *nuoB-SPA*::*kan*	[77]
DY330 *nuoCD-SPA*::*kan*	DY330, *nuoCD-SPA*::*kan*	[77]
DY330 *nuoE-SPA*::*kan*	DY330, *nuoE-SPA*::*kan*	[77]
DY330 *nuoF-SPA*::*kan*	DY330, *nuoF-SPA*::*kan*	[77]
DY330 *nuoG-SPA*::*kan*	DY330, *nuoG-SPA*::*kan*	[77]
DY330 *sdhA-SPA*::*kan*	DY330, *sdhA-SPA*::*kan*	[77]
DY330 *sdhB-SPA*::*kan*	DY330, *sdhB-SPA*::*kan*	[77]
DY330 *cyoB-SPA*::*kan*	DY330, *cyoB-SPA*::*kan*	[77]
DY330 *cyoC-SPA*::*kan*	DY330, *cyoC-SPA*::*kan*	[77]
DY330 *nuoA-SPA*::*kan ΔviaA*::*cat*	DY330, *nuoA-SPA*::*kan*, *ΔviaA*::*cat*	This paper
DY330 *nuoCD-SPA*::*kan ΔviaA*::*cat*	DY330, *nuoCD-SPA*::*kan*, *ΔviaA*::*cat*	This paper
DY330 *nuoF-SPA*::*kan ΔviaA*::*cat*	DY330, *nuoF-SPA*::*kan*, *ΔviaA*::*cat*	This paper
Hfr Cavalli (Hfr C)	Hfr(PO2A), *relA1*, *spoT1*, *metB1*, *rrnB-2*, *mcrB1*, *creC510*	[78]
Hfr C *ΔravA*::*cat* *	Hfr C, *ΔravA*::*cat*	This paper
Hfr C *ΔviaA*::*cat*	Hfr C, *ΔviaA*::*cat*	This paper
Hfr C *ΔravA-viaA*::*cat*	Hfr C, *ΔravAviaA*::*cat*	This paper

Plasmids	Description	Reference
p11	Cloning vector derived from pET15b(+)	[79]
pR	p11-*ravAp-ravA*, for overexpression of RavA regulated by the native *ravA* promoter	This paper
pR_K52Q_	p11-*ravAp-ravA(K52Q)*, for overexpression of RavA Walker A mutant regulated by the native *ravA* promoter	This paper
pRV	p11-*ravAp-ravAviaA*, for RavA and ViaA overexpression regulated by the native *ravA* promoter	This paper
pR_K52Q_V	p11-*ravAp-ravA(K52Q)viaA*, for overexpression of RavA Walker A mutant and wild-type ViaA regulated by the native *ravA* promoter	This paper

*cat* = chloramphenicol acetyltransferase gene; confers resistance to chloramphenicol.

*kan* = kanamycin resistance gene.

ViaA expression is increased in *ΔravA*::*cat* compared to WT (see Figure S2).

For the customized *E. coli* synthetic genetic arrays (eSGA) [26], *ΔravA*::*cat*, *ΔviaA*::*cat* and *ΔravAviaA*::*cat*, were generated by transducing the *cat* KO cassettes from MG1655 into the Hfr C background via P1 bacteriophage as described [24]. For immunoprecipitation, DY330 strains expressing endogenous proteins fused with a C-terminal SPA (Sequential Peptide Affinity) tag for NuoA, NuoB, NuoCD, NuoE, NuoF, NuoG, SdhA, SdhB, CyoB and CyoC were made as described [27]. In addition, *ΔviaA*::*cat* equivalents were also constructed for the strains expressing NuoA-SPA, NuoCD-SPA and NuoF-SPA via P1 phage transduction [24].

All plasmids used are also listed in Table 1. The vector p11 was obtained from the Toronto Structural Genomics Consortium (SGC). The plasmids p11-*ravAp-ravA* (pR) and p11-*ravAp-ravAviaA* (pRV) were constructed by cloning the *ravA* or the *ravAviaA* open reading frame (ORF) along with the native *ravA* promoter (*ravAp*; 206 bp immediately upstream of the *ravA* ORF) into the p11 plasmid. The PCR primers RAVA2_forward (5′-gtggatccgaaatgtgtgcttagtcccttg-3′) and RAVA_reverse (5′-tacgtaggatccttagcattgttgtgcctggcg-3′) were used to amplify the required DNA fragment for the pR plasmid, and the primers RAVA2-forward and VIAA_reverse (5′-ctatggatccttatcgccgccagcgtctgagc-3′) for the pRV plasmid. All fragments were cloned into p11 using the BglII and BamHI restriction sites, which removed the endogenous T7 promoter sequence in the process. To generate the Walker A mutant of RavA, the point mutation K52Q was introduced to the Walker A motif of RavA (GPPGIAKS; mutated residue is underlined) in both pR and pRV, using the QuikChange Site-Directed Mutagenesis kit (Stratagene) and the primers RavA_K52Q_F (5′-cgccaggtattgcccaaagtttgatcgcc-3′) and RavA_K52Q_R (5′-ggcgatcaaactttgggcaatacctggcg-3′), which yielded the plasmids p11-*ravAp-ravA_K52Q_* (pR_K52Q_) and p11-*ravAp-ravA_K52Q_viaA* (pR_K52Q_V), respectively. All plasmids were verified by DNA sequencing.

### Quantification of RavA and ViaA levels in cells

WT *E. coli* MG1655 cells were grown in Luria-Burtani (LB) media (10 g/L bacto-tryptone, 5 g/L yeast extract, and 10 g/L NaCl) at 37°C aerobically in 2-L culture flasks with vigorous shaking for 24 hours. Cell growth was tracked by monitoring the changes in OD_600_ at specific time points. Cells were harvested every two hours by centrifugation and flash-frozen in liquid nitrogen until use. To determine the levels of RavA and ViaA, cell pellets were thawed on ice and then resuspended in a 0.1 M potassium phosphate buffer (pH 7.5) supplemented with 0.1 M NaCl. The volume of each sample was adjusted to achieve a final cell count of approximately 3.8×10^9^ cells/mL as determined by OD_600_. Cells were lysed by sonication followed by mixing with 4× SDS-PAGE sample buffer (200 mM TrisHCl, pH 6.8, 8% SDS, 0.4% bromophenol blue, 40% glycerol, and 400 mM β-mercaptoethanol), and the proteins were separated on 10% or 12% polyacrylamide gels. The amounts of RavA and ViaA were determined by quantitative Western blotting. The numbers of RavA and ViaA molecules expressed per cell were then calculated based on the molecular weights of the two proteins. For comparison, the level of the ClpP protease was also analyzed, while the inner membrane-bound signal peptidase LepB was used as a loading control. The α-RavA, α-Via and α-ClpP rabbit polyclonal antibodies were generated at the Division of Comparative Medicine, University of Toronto. The α-LepB rabbit polyclonal antibody was a generous gift from Dr. Jan Willem de Gier (Stockholm University, Sweden). Purified RavA, ViaA, and ClpP proteins were used as quantification standards. To estimate the number of proteins per cell, we used the standard conversion assuming 1 OD_600_ = 5×10^8^ cells/mL for *E. coli* cells.

### Subcellular localization of RavA and ViaA

WT *E. coli* MG1655 cells were grown in LB at 37°C for 16–18 hours to stationary phase. Subcellular fractionation of the cells was performed as described in [28] and [29], with the following modification. After the extraction of periplasmic proteins by osmotic shock, cells were spun down by centrifugation at 4°C for 30 minutes. Cells were re-suspended in 20 mM TrisHCl (pH 8.0) supplemented with 2 mM EDTA (pH 8.0), and were lysed by French Press. The cytosolic fraction was then cleared of membrane vesicles by ultracentrifugation at ∼190000× g at 4°C for 1 hour in a Beckman-Coulter Optima TLX bench-top ultracentrifuge. Subcellular localization of RavA and ViaA was then determined by Western blotting. The ClpP protease and the inner membrane-bound LepB signal peptidase were chosen as the localization standards for the cytoplasmic and membrane proteins, respectively. Protein levels were estimated by densitometry using Quantity One v. 4.6.5 (Bio-Rad).

### Microarray experiments and data analysis

MG1655 WT, *ΔravA*::*cat*, WT+p11 and WT+pRV were grown in LB at 37°C with a starting OD_600_ of ∼0.025. Stationary phase cells were harvested when OD_600_ reached ∼3 and total RNA was isolated from 500 µL aliquots of each strain using the Qiagen RNeasy Mini Kit with RNAprotect Bacteria Reagent following the manufacturer's instructions. Samples were stored at −80°C until use. Total RNA quality was assessed using the Agilent 2100 Bioanalyzer (Agilent Technologies).

All the microarray experiments were carried out at the Centre for Applied Genomics Microarray Facility, Hospital for Sick Children (Toronto). Sample preparation and array processing were performed following standard protocols. cDNA synthesis was performed with Invitrogen Superscript II Reverse Transcriptase enzyme using random primers and 10 µg total RNA template. RNA template was subsequently degraded using NaOH, which was followed by cDNA cleanup using Qiagen MinElute PCR Purification Columns. The purified cDNA was fragmented with DNase I (GE Healthcare) and labelled with biotin at the 3′-end using GeneChip DNA Labelling Reagent (Affymetrix) and Terminal Deoxynucleotidyl Transferase (Promega). 2 to 5 µg of biotin-labelled cDNA were used in the subsequent hybridization to the *E. coli* Genome 2.0 Arrays. Hybridization, washing, and staining were performed in the Affymetrix GeneChip Hybridization Oven 640 and Fluidics Station 450. Arrays were scanned using the Affymetrix GeneChip Scanner 3000. Three replicates were prepared for each of the five strains used.

Single array data analysis was performed using the GeneChip Operating Software (GCOS). Array signal intensities were globally scaled using an All Probe Sets Scaling strategy, with a target signal of 150. The presence or absence of signals was determined using default parameters for the GeneChip *E. coli* Genome 2.0 Array. A signal intensity of zero was automatically assigned to any gene considered as ‘absent’. All details pertaining to the statistical analysis of the raw data can be found in the Affymetrix GeneChip Analysis Manual (Data analysis fundamentals; available on the Affymetrix company website). Both raw and per-assay-normalized data were deposited in the ArrayExpress database of the European Bioinformatics Institute (EMBL-EBI) (Accession number: E-MTAB-2001).

Comparison analysis of the resulting data was performed for *ΔravA*::*cat* vs. WT and WT+pRV vs. WT+p11, using a bootstrapping approach for unpaired data. All analyses, based on t-statistics, were performed using in-house software. Changes in gene expression levels having p-values less than 0.05 were considered significant and the signal log_2_ ratio of these changes were calculated. Only significant changes with absolute signal log_2_ ratios of 0.6 (∼1.5 fold absolute change in transcript level) or greater were selected for further analysis. A manual review of the change in gene levels was then performed. All remaining genes were examined using the data currently available in the databases EcoCyc [30] and UniProt [31], and were grouped together into operons whenever possible. Fold-changes in gene expression are represented as heatmaps that are generated with the online software Matrix2png [32].

### *E. coli* Synthetic Genetic Array (eSGA) analysis

Genes deemed functionally linked to RavA-ViaA by the microarray experiments were validated further by customized *E. coli* Synthetic Genetic Arrays [26], [33]. The double deletion mutants for *ΔravA*::*cat*, *ΔviaA*::*cat* and *ΔravAviaA*::*cat* were constructed via conjugation between the respective Hfr C donor strains carrying the KO cassettes for *ravA* and/or *viaA* and the selected BW25113 recipient strains from the Keio collection of *E. coli* single-gene deletion mutants [34], [35], following the same protocols as described previously in [26] and [33]. The closest flanking genes upstream and downstream of the genes/operons of interest that show no genetic interaction with either *ravA* or *viaA* were used as controls.

### Growth of *E. coli* MG1655 in cultures containing sublethal concentrations of different antibiotics

*E. coli* MG1655 WT, *ΔravA*, *ΔviaA* and *ΔravAviaA* were grown on LB-agar plates overnight at 37°C to obtain single colonies. Pre-cultures were prepared for each strain by inoculating a single colony into 3 mL of fresh LB and grown with rigorous shaking at 37°C overnight. Next day, the pre-cultures were used to inoculate fresh LB supplemented with 4 µg/mL kanamycin, 6 µg/mL streptomycin, 0.5 µg/mL tetracycline, or 1.2 µg/mL chloramphenicol at a starting OD_600_ of ∼0.01. The dosages of antibiotics used were based on similar experiments as reported in [36]. Further supplementation to the growth media included the addition of 750 µM reduced L-glutathione (GSH) or 250 µM 2,2′-dipyridyl (DP) where applicable. Growth of cells was monitored via OD_600_ using a SpectraMax 340PC Plate Reader. Three independent cultures were prepared for each strain and for each growth condition.

Complementation experiments were performed the same way on the following strains: WT transformed with p11, pR, pRV, pR_K52Q_ or pR_K52Q_V; *ΔravA* transformed with p11, pR or pR_K52Q_; and *ΔravAviaA* transformed with p11, pR, pRV or pR_K52Q_V. 100 µg/mL ampicillin was added to the growth media for plasmid maintenance.

### Analysis of intracellular oxidative stress by DHR fluorescence

MG1655 WT+p11, *ΔravAviaA*+p11, *ΔravAviaA*+pRV and *ΔravAviaA*+pR_K52Q_V strains were grown on LB-agar plates supplemented with 100 µg/mL ampicillin overnight at 37°C to obtain single colonies. Pre-cultures were prepared by inoculating fresh LB+50 µg/mL ampicillin and grown overnight at 37°C with rigorous shaking. Next day, the pre-cultures were used to inoculate fresh LB, supplemented with 4 µg/mL kanamycin, 8 mM GSH and/or 250 µM DP as required, at a starting OD_600_ of ∼0.05. Cells were grown at 37°C with rigorous shaking to late log phase (4–5 hours). The membrane-permeable reactive oxygen species (ROS) indicator dihydrorhodamine 123 (DHR) was then added to each culture at 110 µM (8 µg/mL) final concentration from a 5 mg/mL DMSO stock solution, followed by a 30-minute incubation at 37°C without shaking. Cells that were incubated with DMSO instead of DHR were used as unstained controls. Afterwards, cells were harvested by centrifugation and re-suspended in PBS (10 mM Na_2_HPO_4_, 2 mM KH_2_PO_4_, pH 7.4, 137 mM NaCl, and 2.7 mM KCl). 100-µL aliquots of the cell suspensions were then collected in a 96-well plate, and both DHR fluorescence (λ_ex_ = 500 nm; λ_em_ = 530 nm) and OD_600_ were measured using a Perkin Elmer EnSpire 2300 Multi-label Reader. The raw DHR fluorescence readings were normalized by their respective OD_600_ to allow comparison across samples. Autofluorescence was determined from unstained cells and subtracted from the normalized fluorescence readings.

### Suppression mutation analysis to identify direct functional targets of RavA-ViaA

*E. coli* BW25113 single-gene KO's were selected from the Keio collection [34], [35]. Clean KO's were then generated using the pCP20 plasmid as described above. After confirming the removal of the kanamycin resistance KO cassette and the curing of pCP20, each clean KO was transformed with p11, pRV or pR_K52Q_V. The aerobic growth of the transformed clean KOs in LB or LB+4 µg/ml kanamycin was monitored by OD_600_ over 10 hours. Three independent cultures were prepared for each strain tested. To construct the growth profiles for each strain, the data collected for growth in LB+kanamycin were normalized with the corresponding data collected for growth in LB. This is necessary to exclude any inherent differences in growth due to the KO's genetic background that are independent of the effects of RavA-ViaA.

### Identifying physical interactors of RavA-ViaA by immunoprecipitation

To confirm the interaction between RavA-ViaA and its downstream targets identified by suppression mutation analysis, DY330 strains expressing endogenous NuoA, NuoB, NuoCD, NuoE, NuoF, NuoG, SdhA, SdhB, CyoB and CyoC that carry C-terminal SPA tags [27] were grown aerobically or anaerobically in LB at 30°C overnight. Cells were harvested by centrifugation, and then re-suspended in immunoprecipitation (IP) buffer (25 mM TrisHCl, pH 7.5, 100 mM KCl, 10 mM MgCl_2_, 1 mM CaCl_2_, 0.2 mM EDTA, 1% Triton X-100, 10% glycerol, and 0.5 mM DTT) supplemented with 1 mg/mL lysozyme and 0.1 U/mL DNase I. Cells were lysed by sonication, and the cell lysate cleared of insoluble debris by centrifugation at 4°C. The protein complexes carrying the SPA-tagged targets were purified by incubating the cell lysate with α-FLAG M2 affinity gel (Sigma-Aldrich) at 4°C for 1 hour, followed by three 5-minute washes with IP buffer. The bound complexes were eluted using 3×FLAG peptide re-suspended in IP buffer at 1 mg/mL. The complexes were analyzed by SDS-PAGE and Western blotting for the presence of RavA and ViaA.

To assess the role of ViaA in mediating the interaction between RavA and the SPA-tagged targets, *viaA* was deleted (*ΔviaA*::*cat*) in DY330 strains expressing NuoA-SPA, NuoCD-SPA and NuoF-SPA and the immunoprecipitation experiment were repeated.

## Results

### Expression and localization of RavA and ViaA

**Figure 1:**
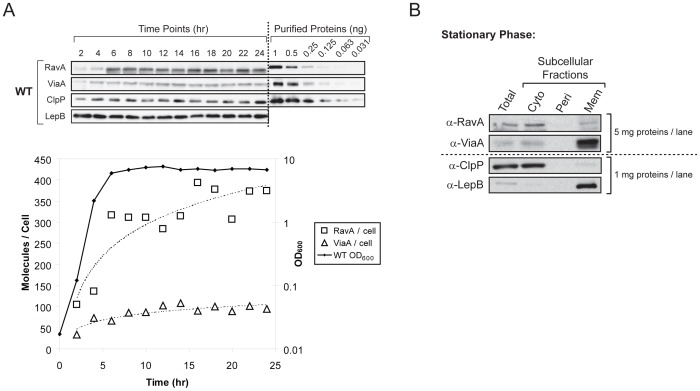
Expression and localization of RavA and ViaA in *E. coli* MG1655. (A) Expression of RavA and ViaA in WT MG1655 grown aerobically in LB at 37°C profiled over 24 hours by quantitative Western blotting. Both ClpP and LepB were used as loading controls. Different amounts of purified RavA, ViaA, and ClpP were used as indicated to provide the necessary quantification standards. Both OD_600_ of the culture and the amount of RavA and ViaA expressed per cell at each time point are shown graphically in the lower panel. Dotted lines trace the expression levels of RavA and ViaA. (B) Total cell lysate and subcellular fractions of WT MG1655 cells grown aerobically to stationary phase in LB at 37°C were Western-blotted for the presence of RavA and ViaA. ClpP and LepB provide localization standards for cytoplasmic and membrane proteins, respectively. The amount of proteins loaded per lane for each blot is as indicated.

In an effort to assess the function of RavA and ViaA in *E. coli*, we first investigated the expression and localization profiles of the two proteins. For aerobically growing culture in LB media at 37°C, the optimal expression of both proteins occurred when cells entered stationary phase (6 hours post inoculation) consistent with our previous observations that the *ravAviaA* operon is induced by σ^S^ [19]. We estimated that approximately 350 molecules of RavA and 90 molecules of ViaA are present per cell at optimum (Fig. 1A). These numbers are considerably lower in comparison to housekeeping proteins such as the molecular chaperone DnaK (11000–12000 molecules per cell [37]), the ClpP subunit of the ClpXP protease complex (approximately 15000 molecules per cell; Fig. S1) or the ribosome-associated trigger factor (approximately 31000 molecules per cell [38]).

At stationary phase, RavA is mainly localized to the cytoplasm, while ViaA is found in both the cytoplasm and unexpectedly, the inner membrane fraction (Fig. 1B). Bioinformatic analysis of ViaA's primary sequence does not reveal any signal peptides or membrane-associating sequence motifs (data not shown). Thus, the apparent localization of ViaA to the cell membrane is likely an indication of its physical association with a membrane-bound target.

### The function of RavA and ViaA is linked to Fe-S cluster assembly and specific respiratory pathways

In order to identify the biological pathways that are functionally linked to RavA and ViaA, we analyzed the gene expression profile of early stationary phase cells having different RavA/ViaA levels using whole-genome microarrays, namely: *ΔravA*::*cat* vs. WT (set 1) and WT+pRV vs. WT+p11 (set 2). The pathways associated with RavA and ViaA were determined using the genes and operons with statistically significant changes in expression upon manipulation of RavA and ViaA levels. It should be noted that the *ΔravA*::*cat* strain is a *ravA* KO as well as a ViaA overexpressor (Fig. S2), presumably due to a polar effect of the marker on *viaA* transcription. Such a polar effect is not observed if the marker is removed (Fig. S2).

Among a total of 300 different genes showing significant changes in expression in sets 1 or 2 (see Table S1), 7 respond to both the loss and increase in RavA-ViaA levels, i.e. their mRNA levels change in both sets 1 and 2, namely: *asnA*; *cysC* and *cysD*; *feoA*, *feoB* and *feoC*; and *metK*. For the genes whose mRNA levels change only in set 1 or set 2, many of them are encoded on the same operons, while others share common biochemical pathways (Table S1). Some of these genes have potentially greater functional relevance and thus were examined further (see below), and their organization into operons and/or regulons is illustrated in Fig. S3.

**Figure 2:**
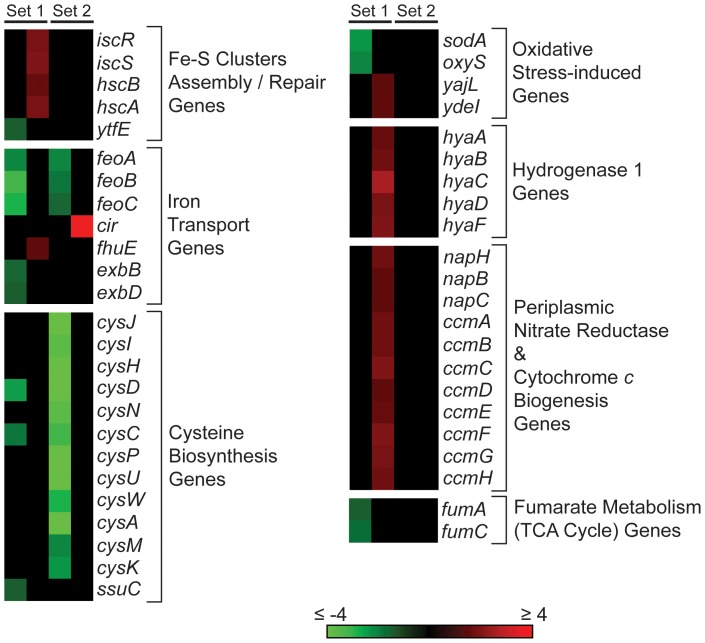
Schematic representation of genes showing significant changes in transcript levels as a result of the deletion or overexpression of RavA/ViaA. Only genes that are functionally relevant to Fe-S clusters assembly and bacterial respiration are shown. Genes that belong to the same functional category are clustered together. In addition, genes that share a common operon are listed, from top to bottom, in the same order as they would appear in the 5′-to-3′ direction within the *E. coli* genome. Changes in gene transcription are represented as heatmaps generated using Matrix2png [32] expressed as fold-changes with respect to either WT for *ΔravA*::*cat* (Set 1), or WT+p11 for WT+pRV (Set 2).

There are 25 genes in both sets 1 and 2 that are associated with the assembly of Fe-S clusters (Fig. 2). These include genes involved in iron uptake and cysteine biosynthesis. In addition, *iscR*, *iscS*, *hscA* and *hscB* (see ‘Fe-S Clusters Assembly/Repair Genes’ in Fig. 2) encode key proteins of the Isc Fe-S clusters assembly pathway [39], while *ytfE* gene encodes a di-iron protein important for the repair of oxidative stress-damaged Fe-S cluster proteins [40].

Several genes related to oxidative stress response were also identified (see ‘Oxidative Stress-induced Genes’ in Fig. 2). These include *sodA* that encodes one of the three superoxide dismutases [41] and *oxyS* that encodes a regulatory small RNA for oxidative stress response [42]. Furthermore, *yajL* (also known as *thiJ*) encodes a chaperone that is involved in oxidative stress response [43], and *ydeI* is important for hydrogen peroxide tolerance [44].

Other genes identified are associated with different respiratory processes. *fumA* and *fumC* (see ‘Fumarate Metabolism Genes’ in Fig. 2) encode two of the three fumarase isozymes found in *E. coli*, which share the same function in converting (*S*)-malate to fumarate in the TCA cycle [45]. FumA is an Fe-S cluster protein and is expressed during aerobiosis, whereas FumC is iron-independent and is induced primarily under oxidative stress conditions [46]. *hyaA*, *hyaB* and *hyaC* (see ‘Hydrogenase 1 Genes’ in Fig. 2) encode the small, large and cytochrome *b* subunits, respectively, of hydrogenase 1, which drives the respiratory hydrogen uptake in the presence of oxygen [47]. The maturation process of hydrogenase 1 requires the accessory proteins encoded by *hyaD* and *hyaF* (see ‘Hydrogenase 1 Genes’ in Fig. 2) [48], [49]. The genes *napH*, *napB* and *napC* (see ‘Periplasmic Nitrate Reductase & Cytochrome *c* Biogenesis Genes’ in Fig. 2) encode three of the five subunits of the periplasmic nitrate reductase (Nap) complex [50], [51]. In this case, NapH is the Fe-S cluster subunit of the Nap complex [51]. Finally, the *ccm* genes (see ‘Periplasmic Nitrate Reductase & Cytochrome *c* Biogenesis Genes’ in Fig. 2) share the same operon as the *nap* genes, and encode proteins that are involved in the biogenesis of *c*-type cytochromes [52]. Although they do not directly participate in bacterial respiration, the Ccm proteins are required for the Nap complex and others that require periplasmic *c*-type cytochromes for their function [52], [53].

To further confirm the microarray study results, we carried out genetic lethal interaction analysis that was recently developed for *E. coli* (eSGA) [26], [33]. To construct the customized eSGA arrays, specific single-gene KO mutants from the Keio collection [34], [35] were selected based on the genes shown in Fig. 2. Genes from the adjacent regions upstream and downstream of the genes being investigated were used as controls. As shown in Fig. S4, the *isc-hsc-fdx*, *cys* and *nap-ccm* operons all exhibited synthetic lethal interactions with *ravA*/*viaA*.

Taken together, both the microarray and eSGA indicated close functional links between RavA-ViaA and the homeostasis of Fe-S cluster proteins as well as bacterial respiration: from the acquisition of required substrates and the assembly of Fe-S clusters to the expression of specific respiratory enzyme complexes that depend on Fe-S cluster proteins for function. Next, we aimed to identify the potential target(s) of RavA-ViaA activity.

### RavA and ViaA sensitize *E. coli* to aminoglycosides

**Figure 3:**
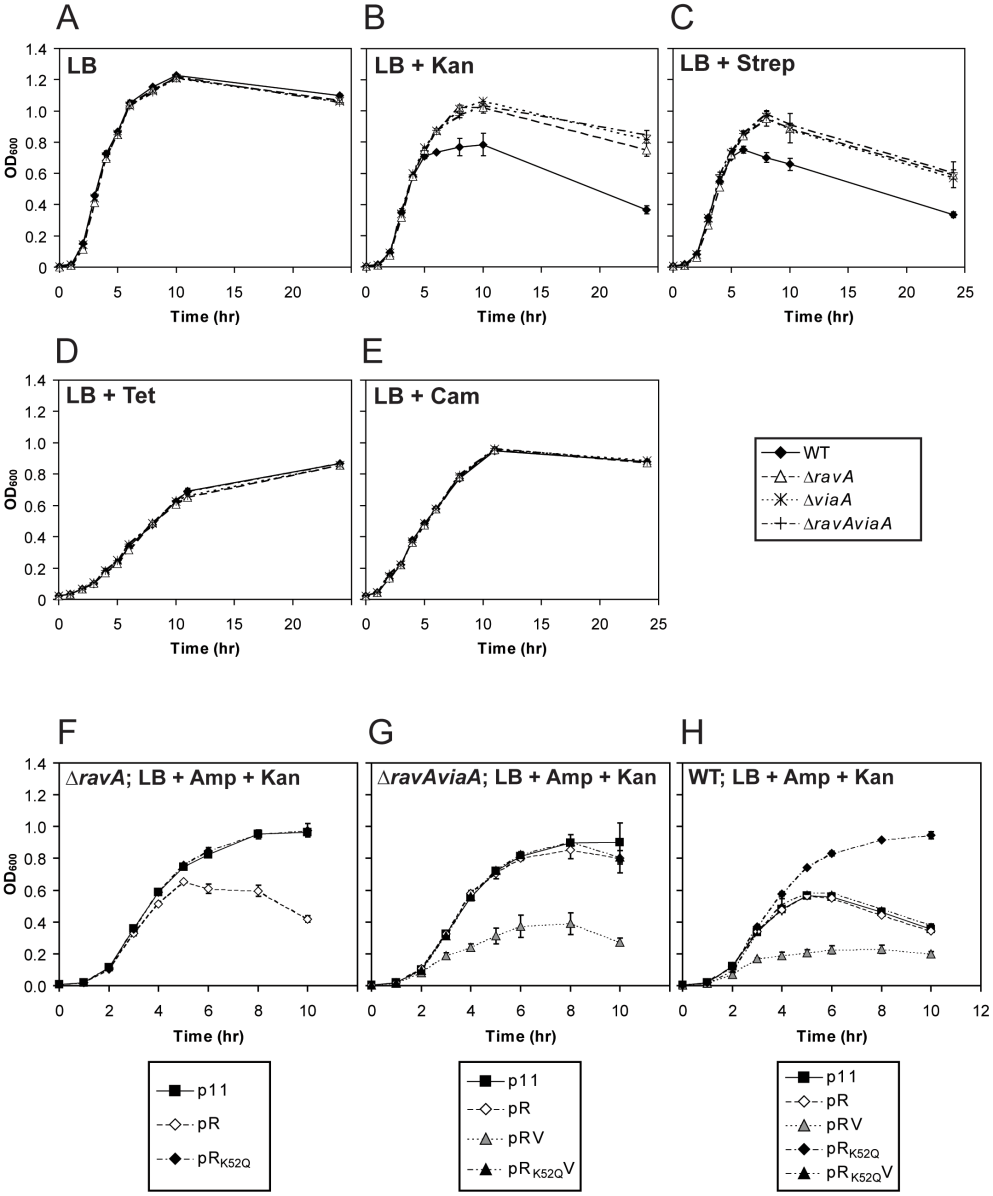
Growth profiles of cells in the presence of sublethal concentrations of aminoglycosides. Growth profiles for MG1655 WT and the KO mutants *ΔravA*, *ΔviaA* and *ΔravAviaA* grown aerobically in LB at 37°C over 24 hours. Growth of cells was monitored using OD_600_ readings at the designated time points. The cultures were supplemented as follows: (A) no antibiotics; (B) 4 µg/mL kanamycin; (C) 6 µg/mL streptomycin; (D) 0.5 µg/mL tetracycline; and (E) 1.2 µg/mL chloramphenicol. To confirm the phenotypes observed, *ΔravA* (F), *ΔravAviaA* (G) and WT cells (H) were complemented with the plasmids p11 (empty vector control), pR, pRV, pR_K52Q_, or pR_K52Q_V. All cultures in the complementation experiments were supplemented with 4 µg/mL kanamycin for stress induction, and 100 µg/mL ampicillin for plasmid maintenance. Error bars were derived from three independent cultures for each strain and for each condition. Details on the *E. coli* strains and plasmids used are given in Table 1.

In a recent whole-genome study, both *ravA* and *viaA* were implicated in sensitizing *E. coli* cells to the presence of sublethal concentrations of aminoglycosides [36]. Notably, a large majority of genes that also confer aminoglycoside sensitivity are involved in Fe-S clusters biogenesis and aerobic respiration [36]. This closely resembles the results of our high-throughput studies discussed above. To validate the deleterious effects of RavA and ViaA on cell growth in the presence of aminoglycosides, we monitored the aerobic growth of WT, *ΔravA*, *ΔviaA* and *ΔravAviaA* (KOs with marker removed) in LB at 37°C. The levels of RavA or ViaA is unchanged if *viaA* or *ravA* is deleted, respectively (Fig. S2). The strains exhibit similar growth behaviour in the absence of antibiotics (Fig. 3A). In the presence of the aminoglycosides kanamycin or streptomycin (Fig. 3B and C), the log-phase growth rate is the same for all the strains, but WT cells reach a lower density of cells in stationary phase compared to the KO cells. This coincides with the fact that RavA-ViaA levels are maximal in early stationary phase. The phenotype is unique to the use of aminoglycosides since WT and the KO strains show similar growth curves when other translation-inhibiting antibiotics, such as tetracycline (Fig. 3D) and chloramphenicol (Fig. 3E), are used.

To further confirm the role of RavA-ViaA in this phenotype, *ΔravA* and *ΔravAviaA* as well as WT were transformed with plasmids carrying the respective genes under the control of the native promoter for the *ravAviaA* operon. The strains have similar growth curves in the absence of aminoglycosides (not shown). For *ΔravA*, complementation with the pR plasmid restores the cell's sensitivity to kanamycin (Fig. 3F). Importantly, no effect is observed when the plasmid carrying the ATPase inactive RavA mutant having the K52Q mutation in the Walker A motif, pR_K52Q_, is used instead of pR (Fig. 3F). The K52Q mutation replaces the highly conserved lysine residue in the Walker A motif that is crucial for the binding and subsequent hydrolysis of ATP [54], [55]. The same type of mutation was used to abolish the ATPase activity in ClpX, the AAA+ component of the ClpXP protease complex [56]. This highlights the importance of RavA's ATPase activity in sensitizing the cells to aminoglycosides.

For *ΔravAviaA*, complementation with pR does not re-sensitize the cells to kanamycin (Fig. 3G). Complementation with pRV re-sensitizes the cells (Fig. 3G), while complementation with the pR_K52Q_V plasmid does not (Fig. 3G). Thus, both RavA and ViaA are needed for the phenotype, and RavA's ATPase activity is also required. Interestingly, the pRV plasmid produces a much stronger sensitization effect on *ΔravAviaA* than the pR plasmid on *ΔravA* (Fig. 3F and G). Given that ViaA expression is unchanged between *ΔravA* and WT (Fig. S2) and that complementation of *ΔravAviaA* with pRV results in a higher ViaA level than its endogenous expression in WT (Fig. S2), we conclude that the manifestation of this phenotype requires RavA's ATPase activity, with ViaA as a potential regulator of RavA's function.

To investigate this issue further, WT cells were transformed with plasmids used in the complementation experiments. WT+pR was found to have the same sensitivity towards kanamycin as WT+p11 (empty vector control) (Fig. 3H), unlike what is observed for *ΔravA*+pR versus *ΔravA*+p11 (Fig. 3F); in contrast, WT+pRV is more sensitive to kanamycin (Fig. 3H). Importantly, the endogenous expression of ViaA is the same in both WT and *ΔravA*, and is unaffected by the presence of p11 or pR (Fig. S2). Hence, the overexpression of RavA alone is indeed insufficient to increase the cell's sensitivity towards aminoglycosides, if ViaA levels remain unchanged. Finally, we found that WT+pR_K52Q_ has the same growth profile as the KO mutants of *ravA* and/or *viaA* (Fig. 3F, G and H). However, WT+pR_K52Q_V is sensitive to kanamycin (Fig. 3H). Evidently, the desensitization effect by RavA_K52Q_ expression is probably caused by the Walker A mutant out-competing its WT counterpart for interaction with ViaA, which manifests into a dominant negative phenotype. This again highlights the critical role of ViaA and of RavA's ATPase activity in this phenotype.

### The RavA-ViaA phenotype is abolished by reduced glutathione and 2,2′-dipyridyl

The exact mechanism behind the bactericidal effects of aminoglycosides remains in dispute. Nevertheless, published works by several different groups on this subject all share the following observations in common: (I) the presence of thiourea (a reducing agent) and/or iron chelators in the growth media increases the cell's tolerance to aminoglycosides; (II) the presence of aminoglycosides induces the *in vivo* oxidation of a fluorescent dye such as hydroxyphenyl fluorescein (HPF) or dihydrorhodamine 123 (DHR) [57]–[62].

**Figure 4:**
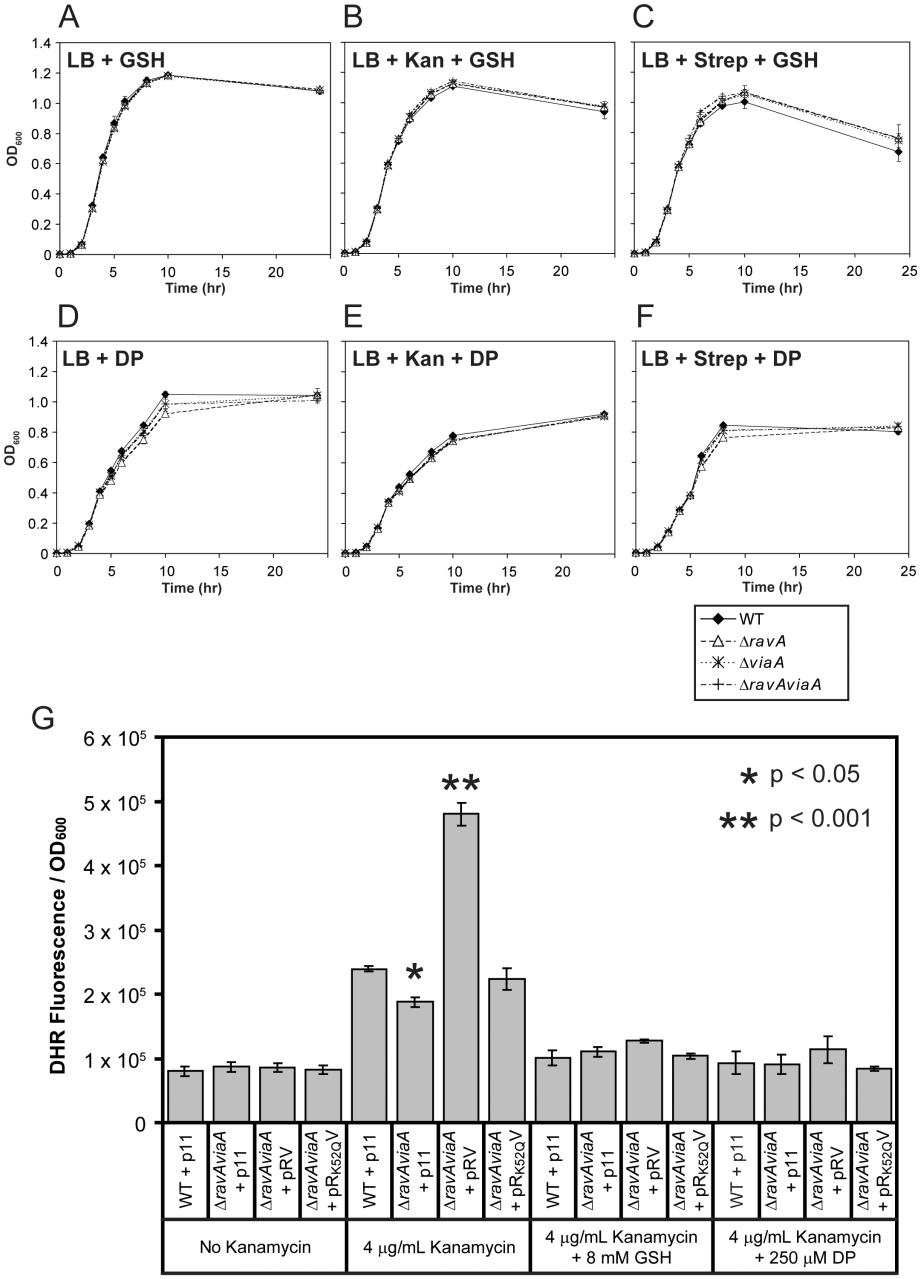
Effects of glutathione and 2,2′-dipyridyl on the growth profiles of cells in the presence of sublethal concentrations of kanamycin. Growth profiles of MG1655 WT and the KO mutants *ΔravA*, *ΔviaA* and *ΔravAviaA* grown aerobically in LB at 37°C without (A, and D) or with kanamycin (B, and E) or streptomycin (C and F). Selected cultures were further supplemented with 750 µM of GSH (A–C) or 250 µM DP (D–F). Kanamycin was added at 4 µg/mL final concentration. Error bars were derived from three independent cultures for each strain and for each condition. In some instances, the error bars are smaller than the symbols used and cannot be seen. (G) DHR fluorescence measurements normalized by OD_600_ for MG1655 WT+p11, *ΔravAviaA*+p11, *ΔravAviaA*+pRV and *ΔravAviaA*+pR_K52Q_V grown aerobically to late log phase in LB at 37°C supplemented with 4 µg/mL kanamycin in the presence or absence of 8 mM GSH or 250 µM DP. Error bars were derived from three independent cultures for each strain and for each condition. To highlight the statistical significance, the p-values for *ΔravAviaA*+p11 vs. WT+p11 (indicated with *) and *ΔravAviaA*+pRV vs. WT+p11 (indicated with **), in the presence of kanamycin, are given in the upper-right corner of the panel.

To determine if the RavA-ViaA phenotype (Fig. 3) relies on the same or a similar mechanism, WT and KO mutants of *ravA* and/or *viaA* were grown in the presence of kanamycin or streptomycin supplemented with reduced glutathione (GSH) or 2,2′-dipyridyl (DP). GSH is a natural antioxidant utilized by *E. coli* [63], while DP is a membrane-permeable chelator that sequesters free intracellular Fe^2+^ ions [64]. The presence of GSH (Fig. 4A–C) or DP (Fig. 4D–F) in the media can effectively rescue the growth reduction of WT cells when exposed to kanamycin (Fig. 4B and E) or streptomycin (Fig. 4C and F), although their effects on the KO mutants of *ravA* and/or *viaA* are minimal by comparison.

In a second experiment, WT+p11, *ΔravAviaA*+p11, *ΔravAviaA*+pRV and *ΔravAviaA*+pR_K52Q_V grown in the presence of kanamycin were all treated with DHR (Fig. 4G). DHR is a membrane-permeable compound that becomes fluorescent and loses membrane permeability when oxidized. It is commonly used as a probe for intracellular ROS [65], although its specificity for ROS detection has recently been questioned in some studies [62], [66], [67]. Nevertheless, as shown in Fig. 4G, without kanamycin, only background levels of DHR fluorescence are detectable among the four strains of cells, showing that the activity of RavA and ViaA do not contribute to DHR oxidation. However, with kanamycin, WT+p11, *ΔravAviaA*+p11, and *ΔravAviaA*+pRV show 2.9-, 2.2- and 5.6-fold increase in DHR fluorescence, respectively. Furthermore, to highlight the importance of RavA's ATPase activity, *ΔravAviaA*+pR_K52Q_V results in only a 2.7-fold increase in DHR fluorescence, resembling WT+p11. As before, the inclusion of either GSH or DP in the media reduces DHR fluorescence in all four strains to background levels. It should also be noted that, in the presence of kanamycin, *ΔravAviaA*+p11 exhibits lower DHR fluorescence than WT+p11 (p<0.05).

Taken together, our results recapture the repeatedly observed effects of antioxidants and iron chelators in conferring greater resistance to the cell against aminoglycosides. This supports the proposition that RavA-ViaA are involved in sensitizing *E. coli* cells to the presence of sublethal concentrations of aminoglycosides.

### RavA-ViaA targets specific Nuo subunits and other respiratory proteins in sensitizing *E. coli* to kanamycin

**Figure 5:**
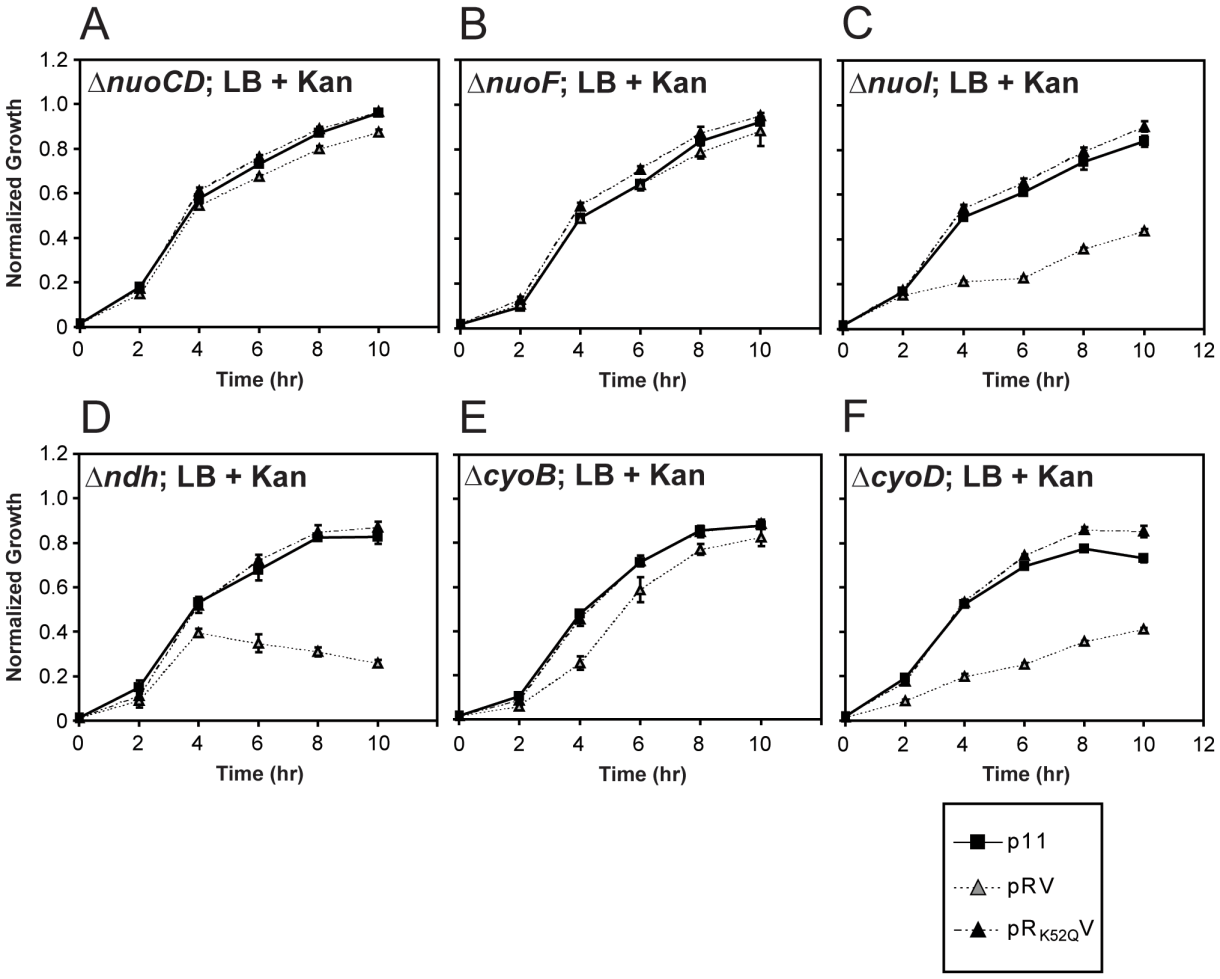
Growth profiles of selected single-gene knockouts. Growth profiles of *ΔnuoCD* (A), *ΔnuoF* (B), *ΔnuoI* (C), *Δndh* (D), *ΔcyoB* (E) and *ΔcyoD* (F) transformed with p11, pRV or pR_K52Q_V plasmids as indicated. To account for the inherent differences in growth rates among the knockouts that are independent of the effects of RavA and ViaA, all growth data collected in the presence of kanamycin were normalized by the corresponding data collected in the absence of the antibiotic.

In order to reveal the direct functional targets of RavA and ViaA, we performed suppression mutation analysis to identify genes that are necessary for the RavA-ViaA phenotype. More specifically, we identified genes whose KO completely abolished the growth suppression induced by RavA-ViaA overexpression in the presence of sublethal concentrations of kanamycin. Candidate genes were chosen based on the results of our high-throughput studies (Fig. 2 and Fig. S4) and the work of Girgis *et al.* [36]. A complete list of genes that were tested is given in Table 2. Six genes were identified: *nuoB*, *nuoCD*, *nuoF*, and *nuoM* that encode 4 of the 13 subunits of NADH:ubiquinone oxidoreductase I (Nuo complex); *sdhB* that encodes the Fe-S cluster subunit of succinate dehydrogenase (Sdh complex); and *cyoB* that encodes subunit I of the cytochrome *b_0_* terminal oxidase (Cyo complex). Examples of the growth profiles of these KO strains are shown for *ΔnuoCD*, *ΔnuoF* and *ΔcyoB* (Fig. 5A, B and E, respectively). In all cases, the overexpression of RavA-ViaA fails to sensitize the cell to kanamycin, unlike what was previously observed in *ΔravAviaA* or WT (Fig. 3G and H).

**Table 2:** Suppression mutation analysis for the RavA-ViaA overexpression-induced sensitization to kanamycin in *E. coli* MG1655.

KO mutations that suppress the RavA-ViaA-overexpression phenotype		
Strain	Gene Product Description/Function*	Associated Cofactors*
*ΔnuoB*	NADH:ubiquinone oxidoreductase I; cytoplasmic subunit B	4Fe-4S
*ΔnuoCD*	NADH:ubiquinone oxidoreductase I; cytoplasmic subunit CD	
*ΔnuoF*	NADH:ubiquinone oxidoreductase I; cytoplasmic subunit F	FMN, 4Fe-4S
*ΔnuoM*	NADH:ubiquinone oxidoreductase I; membrane subunit M	
*ΔsdhB*	Succinate dehydrogenase; Fe-S cluster subunit	2Fe-2S; 4Fe-4S; 3Fe-4S
*ΔcyoB*	Cytochrome *b_0_* terminal oxidase; subunit I	Cytochromes b_562_, b_555_; Cu^2+^

KO mutations with no effect on the RavA-ViaA-overexpression phenotype		
Strain	Gene Product Description/Function	Associated Cofactors
*ΔnuoA*	NADH:ubiquinone oxidoreductase I; membrane subunit A	
*ΔnuoE*	NADH:ubiquinone oxidoreductase I; cytoplasmic subunit E	2Fe-2S
*ΔnuoG*	NADH:ubiquinone oxidoreductase I; cytoplasmic subunit G	2Fe-2S; 3× 4Fe-4S
*ΔnuoH*	NADH:ubiquinone oxidoreductase I; membrane subunit H	
*ΔnuoI*	NADH:ubiquinone oxidoreductase I; cytoplasmic subunit I	2× 4Fe-4S
*ΔnuoJ*	NADH:ubiquinone oxidoreductase I; membrane subunit J	
*ΔnuoK*	NADH:ubiquinone oxidoreductase I; membrane subunit K	
*ΔnuoL*	NADH:ubiquinone oxidoreductase I; membrane subunit L	
*ΔnuoN*	NADH:ubiquinone oxidoreductase I; membrane subunit N	
*Δndh*	Alternative NADH:ubiquinone oxidoreductase II	FAD; Cu^+^; Mg^2+^
*ΔsdhA*	Succinate dehydrogenase; flavoprotein	FAD
*ΔsdhC*	Succinate dehydrogenase; membrane subunit C	Cytochrome b_556_
*ΔsdhD*	Succinate dehydrogenase; membrane subunit D	Cytochrome b_556_
*ΔcyoA*	Cytochrome *b_0_* terminal oxidase; subunit II	
*ΔcyoC*	Cytochrome *b_0_* terminal oxidase; subunit III	
*ΔcyoD*	Cytochrome *b_0_* terminal oxidase; subunit IV	
*ΔiscR*	DNA-binding transcription regulator for Fe-S cluster assembly, biofilm formation & anaerobic respiration	2Fe-2S
*ΔiscS*	Cysteine desulfurase; Isc Fe-S assembly pathway	PLP
*ΔcadA*	Inducible lysine decarboxylase LdcI	PLP
*ΔfeoB*	Fe^2+^ ion uptake transporter	
*ΔcysB*	DNA-binding transcription dual regulator for cysteine biogenesis & novobicin resistance	
*ΔcysI*	Sulfite reductase; hemoprotein subunit	Siroheme; 4Fe-4S
*ΔnadA*	Quinolinate synthase; NAD *de novo* biogenesis	4Fe-4S
*ΔnadB*	L-Aspartate oxidase; NAD *de novo* biogenesis	FAD

Gene annotations were obtained from the online databases EcoCyc [30] and UniProt [31].

Interestingly, among the 24 genes that did not suppress the RavA-ViaA overexpression phenotype, many encode the other subunits of the Nuo, Sdh and Cyo complexes: *ΔnuoI*, *ΔsdhA*, *ΔsdhC*, *ΔsdhD*, *ΔcyoA*, *ΔcyoC* or *ΔcyoD* (Table 2 and Fig. 5). For example, the growth profiles of *ΔnuoI* (Fig. 5C) clearly show that *nuoI* is not needed in facilitating the RavA-ViaA phenotype, yet all Nuo subunits have been shown to be equally important for maintaining full functionality of the Nuo complex [68]. Thus, the functional role of RavA and ViaA appears to extend only to specific subunits or subcomplexes, but not the Nuo complex as a whole. This is further supported by the observation that *Δndh* also fails to suppress the RavA-ViaA phenotype (Fig. 5D), despite the fact that *ndh* encodes an enzyme functionally equivalent to the Nuo complex.

Taken together, these results indicate that RavA and ViaA target only specific subunits of the Nuo, Sdh and Cyo complexes when cells are exposed to sublethal concentrations of aminoglycosides during aerobic growth.

### RavA and ViaA interact with specific Nuo subunits

To obtain conclusive evidence that RavA and ViaA are physically interacting with specific subunits of the Nuo, Sdh and Cyo respiratory proteins in *E. coli*, the genes corresponding to these subunits were endogenously tagged at the 3′ end with a SPA-tag [27], and the tag was used for pull down assays. The SPA-tag consists of three modified FLAG sequences and a calmodulin binding peptide, spaced by a cleavage site for tobacco etch virus protease. The subunits that were successfully tagged are: NuoA, NuoB, NuoCD, NuoE, NuoF, NuoG, SdhA, SdhB, CyoB and CyoC. However, SPA-tagged SdhB and CyoC were not stably expressed and could not be used.

**Figure 6:**
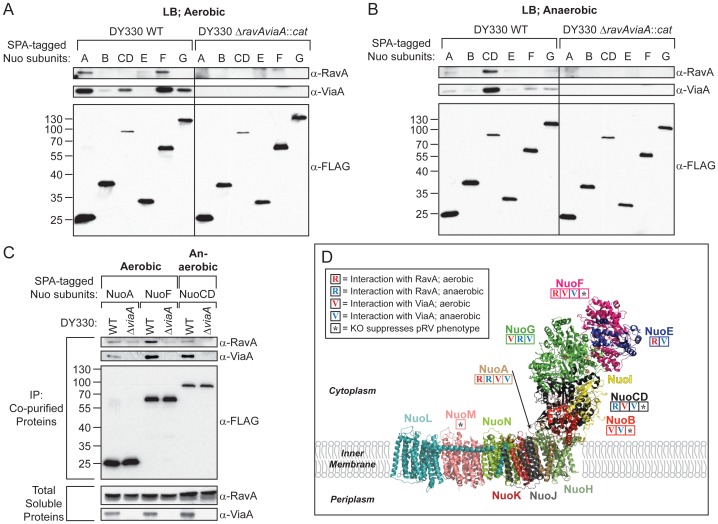
Physical interactions between RavA and ViaA with specific subunits of the Nuo complex under different growth conditions. (A–C) WT, *ΔravAviaA*::*cat*, *ΔviaA*::*cat* DY330 strains having endogenously C-terminally SPA-tagged Nuo subunits were grown to stationary phase in LB under aerobic (A, C) or anaerobic (B, C) conditions. RavA and ViaA that co-purify with the SPA-tagged Nuo subunits were detected by Western blotting using α-RavA and α-ViaA polyclonal antibodies, respectively, whereas the SPA-tagged Nuo subunits were detected using α-FLAG monoclonal antibodies. The *ΔravAviaA*::*cat* strain having the SPA-tagged Nuo subunits is shown (A, B) as a control to confirm the identity of the RavA and ViaA bands detected. While the *ΔviaA*::*cat* strain having the SPA-tagged Nuo subunits is shown (C) to assess the role of ViaA in facilitating the binding of RavA to SPA-tagged NuoA and NuoF under aerobic conditions, and to SPA-tagged NuoC under anaerobic conditions. (D) X-ray structure of the NADH:ubiquinone oxidoreductase I from *Thermus thermophilus* (PDB ID: 3M9S), solved at 3.3 Å [80]. The subunits are identified here using the nomenclature for the *E. coli* NADH:ubiquinone oxidoreductase I (i.e. the Nuo complex) [80], [81]. The subunits Nqo15 and Nqo16 unique to *T. thermophilus* are omitted from the structure for clarity. Physical interactions of specific subunits with RavA and ViaA are indicated by the capital letters R and V, respectively. Red letters denote interactions that were identified in aerobically grown *E. coli*, and blue letters for the interactions in anaerobically grown cells. Asterisks (*) denote the subunits that are necessary for sensitizing the cell to sub-lethal concentrations of kanamycin upon overexpression of RavA and ViaA.

Neither SdhA nor CyoB showed any evidence of physical interaction with RavA and ViaA (data not shown). All of the Nuo subunits tested interacted with RavA and/or ViaA to various degrees (Fig. 6A and B). Since the Nuo complex functions aerobically and anaerobically [69], [70], the pulldowns were carried out under both conditions. In aerobically grown cells, NuoA and NuoF interacted with both RavA and ViaA. NuoE showed weak interaction with only RavA, while NuoB, NuoCD and NuoG all interacted with only ViaA, with NuoB showing weak interaction and NuoG showing moderate interaction (Fig. 6A). NuoCD was not pulled down as efficiently as the other subunits (Fig. 6A). However, in anaerobically grown cells, NuoCD interacted strongly with both RavA and ViaA (Fig. 6B), while the other Nuo subunits exhibited no or weak interactions (Fig. 6B). For all the pulldown assays, control experiments are shown for Nuo tagged strains carrying the *ΔravAviaA* null mutation (Fig. 6A, B). In addition, untagged WT DY330 was also used to rule out unspecific binding (Fig. S5).

Given the primary function of VWA proteins in mediating protein-protein interactions [14], it is possible that the interactions of RavA with the Nuo subunits are mediated by ViaA, which is also found to partly reside on the inner membrane (Fig. 1B). Hence, experiments were repeated using DY330 *ΔviaA*::*cat* strains that express endogenous, C-terminally SPA-tagged NuoA, NuoCD, or NuoF. As shown in Fig. 6C, the absence of ViaA results in significantly decreased binding of all three Nuo subunits to RavA. Neither RavA nor the three SPA-tagged Nuo subunits show any noticeable difference in expression between WT and *ΔviaA*::*cat* strains.

Taken together, these results strongly indicate that the Nuo complex is a functional target of RavA and ViaA, with NuoA and NuoF being the main subunits targeted under aerobic conditions, and NuoCD under anaerobic conditions. Importantly, ViaA is required for mediating the interaction between RavA and the Nuo subunits, which is reflected in both RavA and ViaA being equally important in their sensitization of the cell to aminoglycosides (Fig. 3).

## Discussion

Using a multi-disciplinary approach, we were able to identify novel interactions between RavA-ViaA and specific subunits of the Nuo respiratory complex. A summary of these interactions is illustrated in Fig. 6D. Out of the six Nuo subunits tested for physical interactions, NuoF (aerobically) and the fused NuoCD (anaerobically) showed strong interactions with both RavA and ViaA (Fig. 6A, B) with both Nuo subunits being necessary for RavA and ViaA to sensitize the cells towards kanamycin (Fig. 5A,B). In this regard, it is interesting to note that a recent study on the Nuo proteins in *E. coli* revealed that the inducible lysine decarboxylase LdcI, which we showed to form a large cage-like structure with RavA [19], [20], binds specifically to a variant form of the Nuo complex that lacks NuoL [68]. However, in our pulldown assays (Fig. 6), we did not observe any significant interaction of LdcI with the SPA-tagged Nuo subunits (data not shown). This seems to suggest that an LdcI-RavA-ViaA complex might interact with a specific Nuo subcomplex, when NuoL is deleted. This subcomplex might contain NuoF and NuoCD.

Our phenotypic data suggest that the interaction of RavA-ViaA with NuoF and NuoCD is likely an important part underlying the sensitization of *E. coli* towards aminoglycosides by RavA and ViaA. The exact mechanism behind the bactericidal effects of aminoglycosides is still under debate. Aside from their traditional role in binding ribosomes that causes protein mistranslation [71], one recent model proposes that the bactericidal effects of aminoglycosides may arise from the generation of intracellular reactive oxygen species (ROS) via the Fe^2+^-mediated Fenton reaction [58], [59]. The source of the free Fe^2+^ has been attributed to damaged Fe-S clusters, resulting from increased H_2_O_2_ production caused by the upregulated respiratory activities [58], [59]. However, several groups have recently shown that aminoglycosides can neither increase the level of H_2_O_2_ in the cell nor upregulate bacterial respiration [60], [62], nor is ROS necessary for the bactericidal actions of the antibiotics [62], [72]. Nevertheless, a recent study on the toxicity of protein aggregates generated via aminoglycoside-induced mistranslation has shown that overexpressing AhpF, one of two subunits of the H_2_O_2_ scavenger alkyl hydroperoxide reductase, can effectively increase the cell's tolerance to aminoglycosides by reducing the oxidation and aggregation of mistranslated proteins [73]. This supports the notion that oxidative damage may still play an important role in the cellular toxicity of protein mistranslation. In contrast, the work by Ezraty *et al.* [74] suggests that the bactericidal effect of aminoglycosides is dependent on Fe-S clusters biosynthesis that is independent of ROS. Specifically, the major Isc Fe-S clusters assembly pathway is required for the full maturation and function of the Nuo and Sdh respiratory complexes, which in turn generate proton motive force (PMF) that promotes the uptake of aminoglycosides leading to cell death [74]. The effect of RavA-ViaA might be manifested through such a latter model. Furthermore, the genetic linkage of RavA-ViaA with Fe-S cluster biogenesis genes (Fig. 2) may reflect a chaperone-like role of RavA and ViaA for NuoF, for example, and possibly other Fe-S-carrying targets. The physiological implication of the interaction of RavA-ViaA and possibly LdcI with the Nuo complex is the subject of ongoing studies.

## Supporting Information

Figure S1 **Levels of ClpP in** ***E. coli*** **MG1655.** Expression of ClpP in wild-type (WT) MG1655 grown aerobically in LB at 37°C was profiled over 24 hours by quantitative Western blotting in the same way as RavA and ViaA (see Fig. 1A). Trend lines for the expression of ClpP, RavA, and ViaA are shown as dotted lines.
(TIF)

Figure S2 **Expression levels of RavA and ViaA in various strain backgrounds used in this study.** The various strains of *E. coli* MG1655 as shown were grown aerobically to early stationary phase in LB at 37°C, and the total cell lysate prepared from them were Western-blotted for the presence of RavA and ViaA. The membrane-bound LepB was used as loading control. For WT and the KO mutant strains of *ravA* and/or *viaA*, lysate from ∼7.4×10^7^ cells was loaded per sample, whereas for WT cells transformed with plasmids, lysate from ∼1.3×10^7^ cells was loaded per sample. The tables provide an estimate of the number of RavA and ViaA molecules expressed per cell obtained by densitometry for each strain used. The estimation of RavA and ViaA levels for WT and WT+p11 (indicated by *) was derived from the RavA and ViaA quantification data shown in Fig. 1A.
(TIF)

Figure S3 **Genomic organization of genes relevant to Fe-S clusters assembly or bacterial respiration showing statistically significant changes in the microarray experiments.** Operons of the same regulon involved in the same biochemical pathways are grouped together. The length of the arrow for each gene corresponds to the size of the gene's open reading frame. Transcripts detected in the microarray experiments are highlighted in red, and those that were not detected are in grey. All known transcriptional regulators for each operon are boxed. Activators are indicated with a ‘+’ sign and highlighted in green. Repressors are indicated with a ‘−’ sign and highlighted in red. Dual regulators are indicated with ‘+/−’ and highlighted in orange.
(TIF)

Figure S4 **Genetic interactions between** ***ravA*** **/** ***viaA*** **and genes functionally relevant to Fe-S clusters assembly and bacterial respiration.** Shown are plates demonstrating that the deletion of *ravA*, *viaA*, or *ravAviaA* results in synthetic lethality when genes belonging to the Isc Fe-S assembly, cysteine biosynthesis, or nap-ccm operons are also deleted. Genes sharing the same operon are grouped together in the same row whenever possible. A total of 2 replicates for each of 2 independent colonies were prepared for each donor-recipient pair, and are arranged into a 2×2 configuration as shown. The donors are identified on the left for each row, and the recipients on top of each column. Arrows represent the direction of the genes in each operon (colored in dark grey) relative to the flanking control genes (colored in light grey).
(TIF)

Figure S5 **Immunoprecipitation experiments on WT DY330 and strains expressing SPA-tagged NuoF or NuoCD.** Shown are Western blots for endogenous ViaA and the SPA-tagged NuoF and NuoCD in total soluble proteins (Input) and after immunoprecipitation of the SPA-tagged proteins. DY330 expressing SPA-tagged NuoF under aerobic condition and SPA-tagged NuoCD under anaerobic condition were used. Untagged WT DY330 strain is shown as control.
(TIF)

Table S1 **Genes showing statistically significant changes in transcript levels caused by the deletion or overexpression of RavA/ViaA.** This is a complete list of genes showing significant changes in transcript levels that were detected by the microarray analyses. All changes in expression are shown as fold-changes with respect to WT for *ΔravA*::*cat*, set 1, or WT+p11 for WT+pRV, set 2. Genes showing an increase in expression are listed separately from those showing a decrease. “++” represents a fold-increase that cannot be calculated, and “− −” for a fold-decrease that cannot be calculated, due to the corresponding transcript being undetectable in WT or WT+p11. Genes are sorted by their b-numbers.
(XLS)
