# Supplementary figures and images for: The MoxR ATPase RavA and Its Cofactor ViaA Interact with the NADH:Ubiquinone Oxidoreductase I in Escherichia coli
Source: PLoS One. 2014 Jan 15;9(1):e85529. doi: 10.1371/journal.pone.0085529 (PMC3893208; doi:10.1371/journal.pone.0085529)

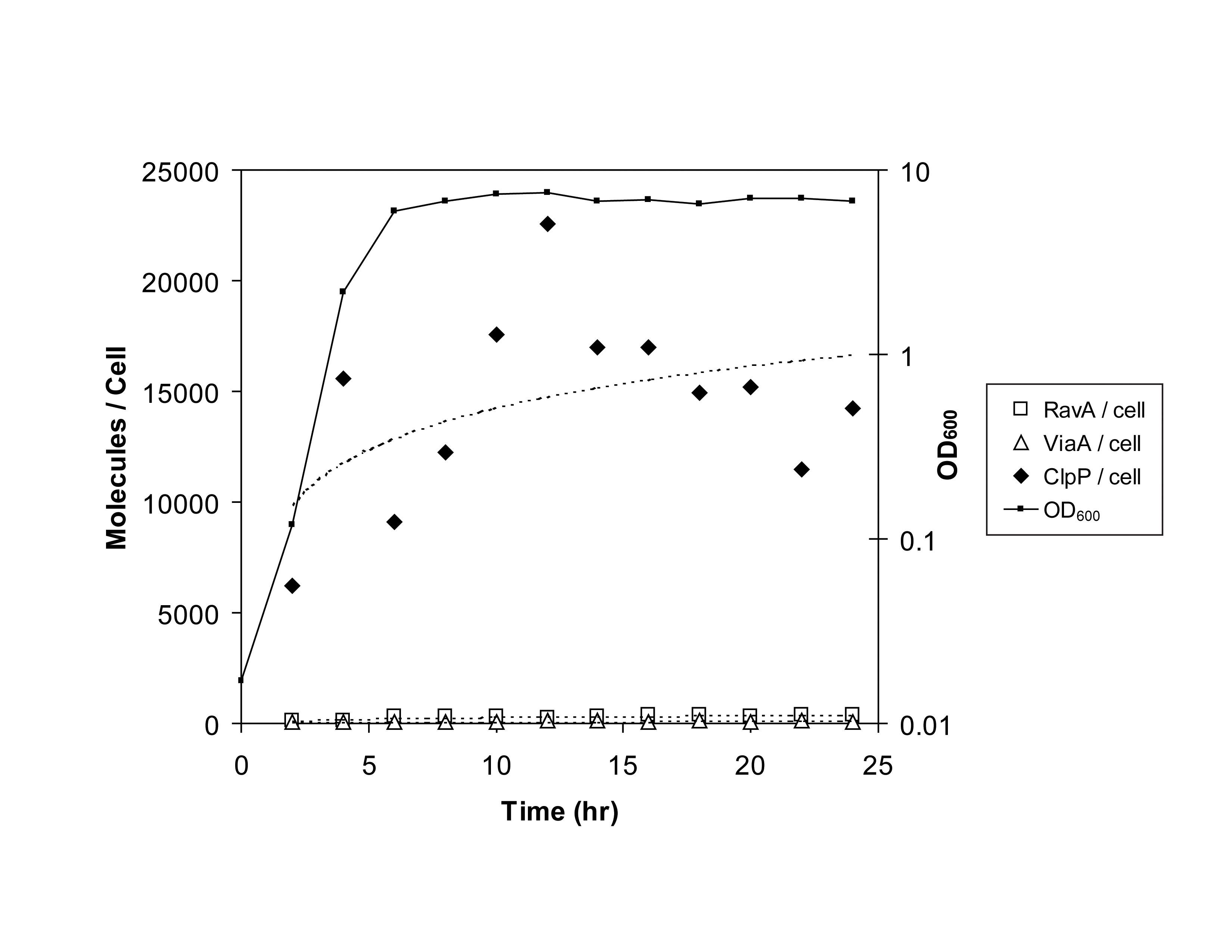

Supplement: Figure S1 — Levels of ClpP in E. coli MG1655. Expression of ClpP in wild-type (WT) MG1655 grown aerobically in LB at 37°C was profiled over 24 hours by quantitative Western blotting in the same way as RavA and ViaA (see Fig. 1A). Trend lines for the expression of ClpP, RavA, and ViaA are shown as dotted lines. (TIF) [file pone.0085529.s001.tif]

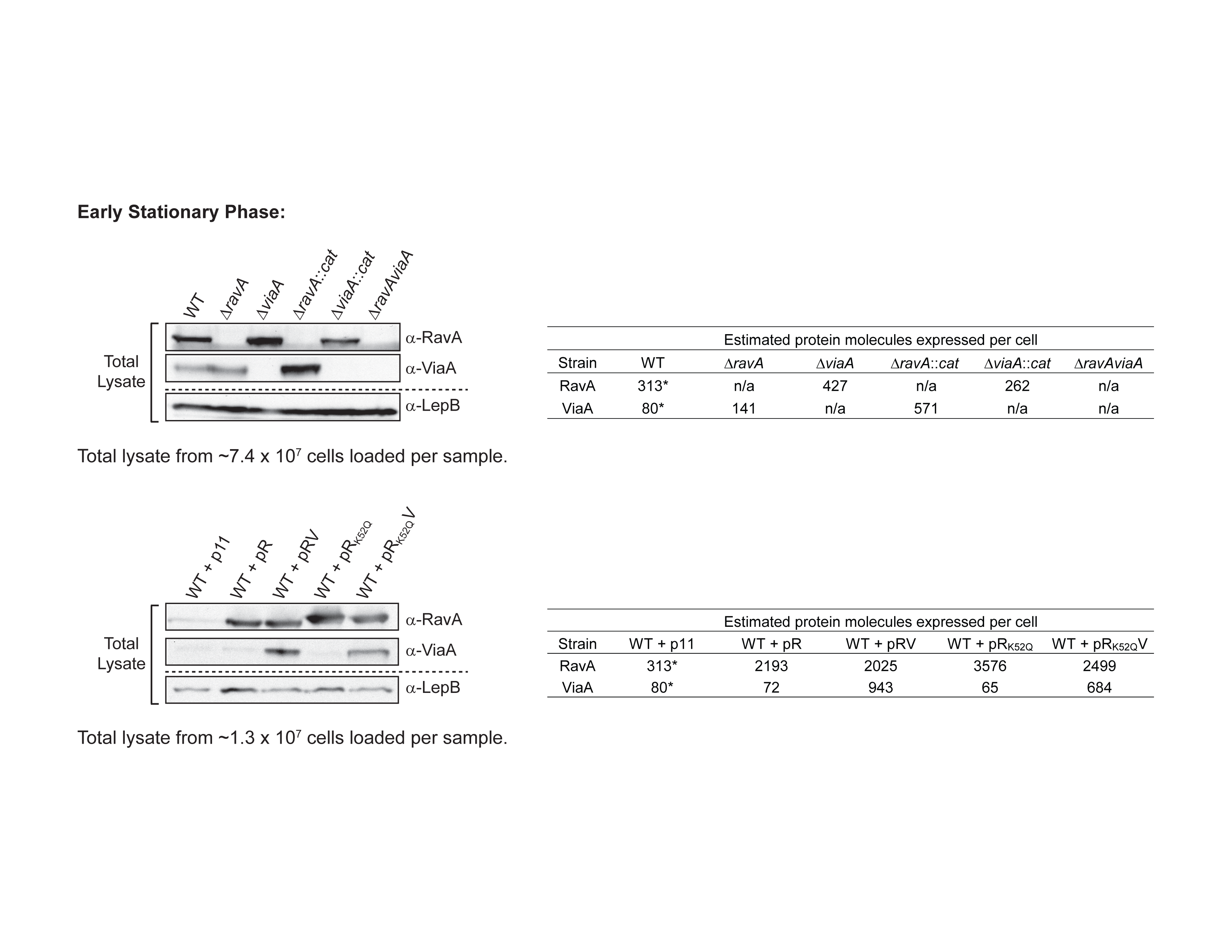

Supplement: Figure S2 — Expression levels of RavA and ViaA in various strain backgrounds used in this study. The various strains of E. coli MG1655 as shown were grown aerobically to early stationary phase in LB at 37°C, and the total cell lysate prepared from them were Western-blotted for the presence of RavA and ViaA. The membrane-bound LepB was used as loading control. For WT and the KO mutant strains of ravA and/or viaA, lysate from ∼7.4×10^7^ cells was loaded per sample, whereas for WT cells transformed with plasmids, lysate from ∼1.3×10^7^ cells was loaded per sample. The tables provide an estimate of the number of RavA and ViaA molecules expressed per cell obtained by densitometry for each strain used. The estimation of RavA and ViaA levels for WT and WT+p11 (indicated by *) was derived from the RavA and ViaA quantification data shown in Fig. 1A. (TIF) [file pone.0085529.s002.tif]

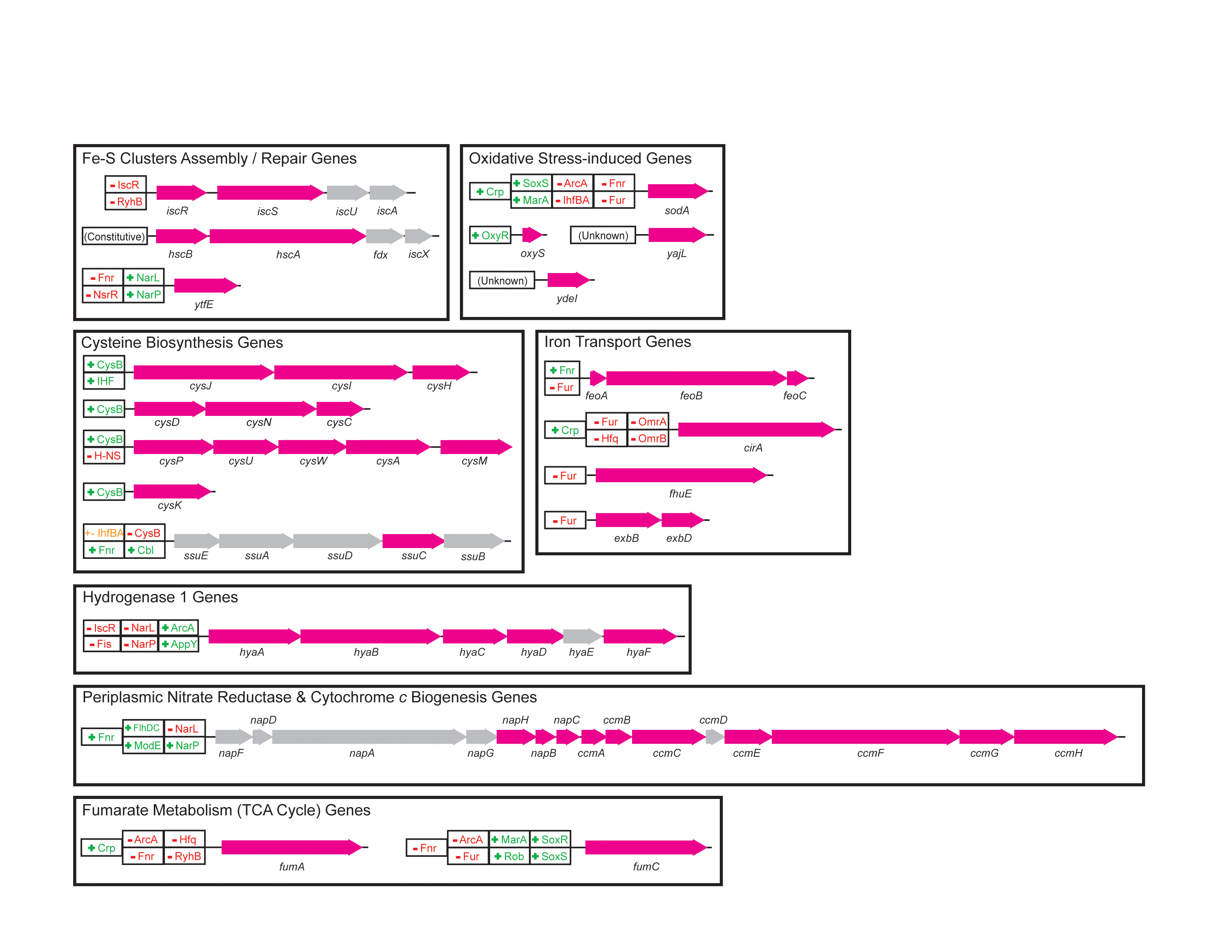

Supplement: Figure S3 — Genomic organization of genes relevant to Fe-S clusters assembly or bacterial respiration showing statistically significant changes in the microarray experiments. Operons of the same regulon involved in the same biochemical pathways are grouped together. The length of the arrow for each gene corresponds to the size of the gene's open reading frame. Transcripts detected in the microarray experiments are highlighted in red, and those that were not detected are in grey. All known transcriptional regulators for each operon are boxed. Activators are indicated with a ‘+’ sign and highlighted in green. Repressors are indicated with a ‘−’ sign and highlighted in red. Dual regulators are indicated with ‘+/−’ and highlighted in orange. (TIF) [file pone.0085529.s003.tif]

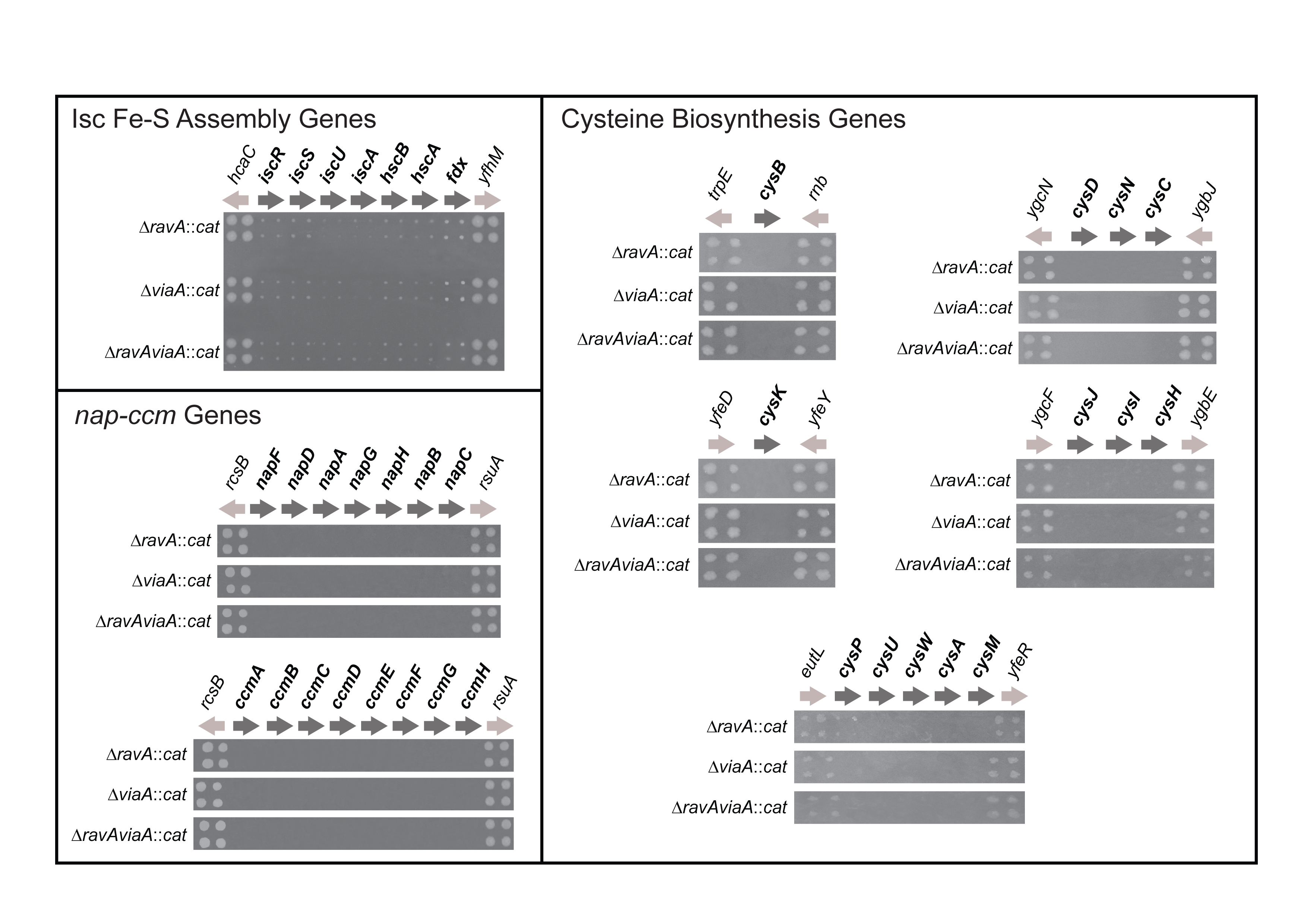

Supplement: Figure S4 — Genetic interactions between ravA / viaA and genes functionally relevant to Fe-S clusters assembly and bacterial respiration. Shown are plates demonstrating that the deletion of ravA, viaA, or ravAviaA results in synthetic lethality when genes belonging to the Isc Fe-S assembly, cysteine biosynthesis, or nap-ccm operons are also deleted. Genes sharing the same operon are grouped together in the same row whenever possible. A total of 2 replicates for each of 2 independent colonies were prepared for each donor-recipient pair, and are arranged into a 2×2 configuration as shown. The donors are identified on the left for each row, and the recipients on top of each column. Arrows represent the direction of the genes in each operon (colored in dark grey) relative to the flanking control genes (colored in light grey). (TIF) [file pone.0085529.s004.tif]

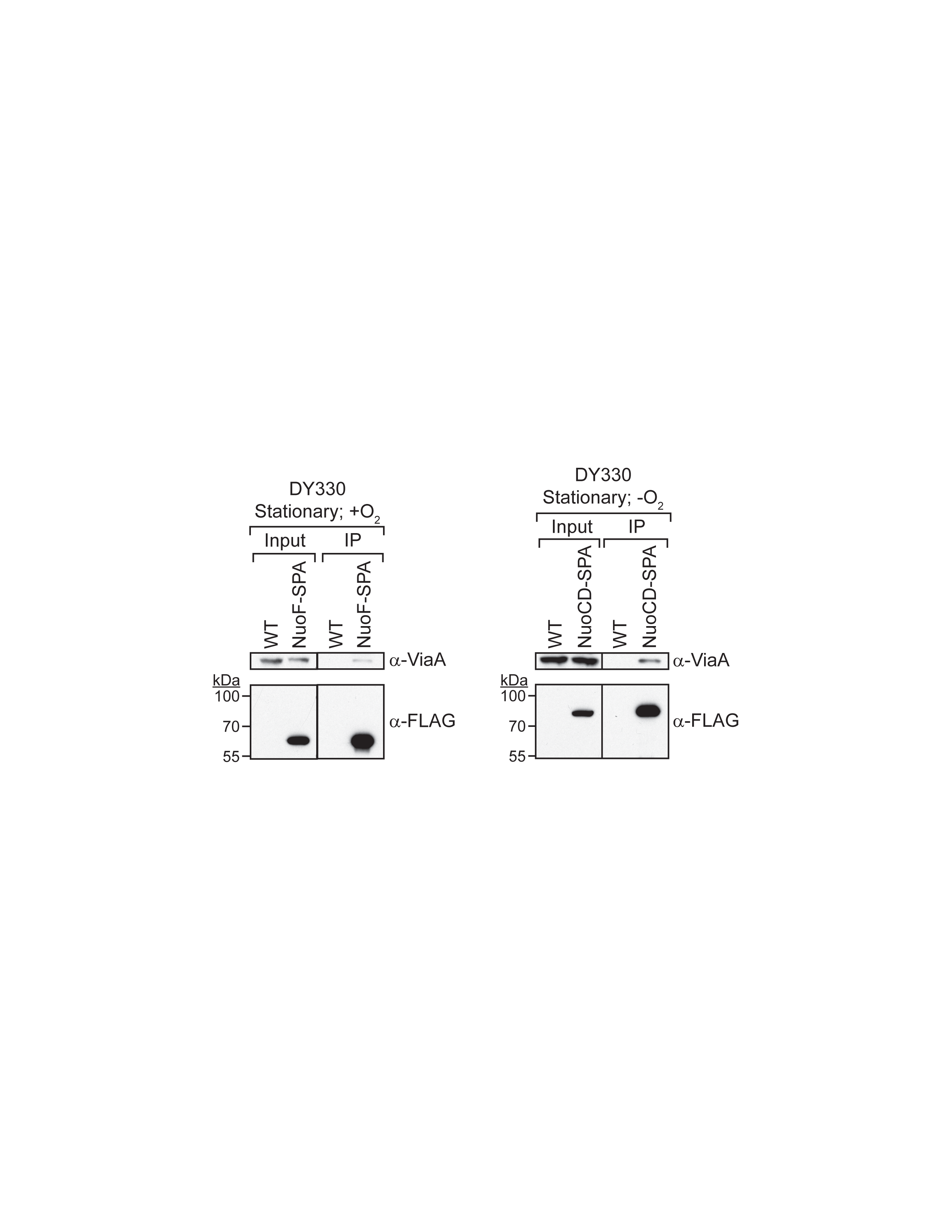

Supplement: Figure S5 — Immunoprecipitation experiments on WT DY330 and strains expressing SPA-tagged NuoF or NuoCD. Shown are Western blots for endogenous ViaA and the SPA-tagged NuoF and NuoCD in total soluble proteins (Input) and after immunoprecipitation of the SPA-tagged proteins. DY330 expressing SPA-tagged NuoF under aerobic condition and SPA-tagged NuoCD under anaerobic condition were used. Untagged WT DY330 strain is shown as control. (TIF) [file pone.0085529.s005.tif]
